# Extraction, Purification, and Elucidation of Six Ginkgol Homologs from *Ginkgo biloba* Sarcotesta and Evaluation of Their Anticancer Activities

**DOI:** 10.3390/molecules27227777

**Published:** 2022-11-11

**Authors:** Fengnan Li, Isaac Duah Boateng, Xiaoming Yang, Yuanyuan Li

**Affiliations:** 1 School of Food and Biological Engineering, Jiangsu University, Zhenjiang 212013, China; 2 Food Science Program, Division of Food, Nutrition and Exercise Sciences, University of Missouri, 1406 E Rollins Street, Columbia, MO 65211, USA; 3 Zhenjiang Food and Drug Supervision and Inspection Center, Zhenjiang 212004, China

**Keywords:** *Ginkgo biloba*, *Ginkgo biloba* sarcotesta, ginkgol homologs, anticancer activity, structure–activity

## Abstract

Ginkgols are active constituents from *Ginkgo biloba* L. (GB) and have pharmacological activities, such as antibacterial and antioxidant activities. In our previous report, only five ginkgols were separated. However, ginkgol C17:1 had two isomers, for which their separation, identification, and bioactivities have not yet been investigated. Hence, this research reports the successful isolation of six ginkgol homologs with alkyl substituents—C17:1-Δ^12^, C15:1-Δ^8^, C13:0, C17:2, C17:1-Δ^10^, and C15:0—for the first time using HPLC. This was followed by the identification of their chemical structures using Fourier transform infrared (FTIR), ultraviolet (UV), gas chromatography and mass spectrometry (GC-MS), carbon-13 nuclear magnetic resonance (^13^C-NMR), and proton nuclear magnetic resonance (^1^H-NMR) analysis. The results showed that two ginkgol isomers, C17:1-Δ^12^ and C17:1-Δ^10^, were obtained simultaneously from the ginkgol C17:1 mixture and identified entirely for the first time. That aside, the 3-(4,5-dimethyl-2-thiazolyl)-2,5-diphenyl-2-H-tetrazolium bromide (MTT) assay showed that the six ginkgol homologs possessed significant antiproliferation effects against HGC and HepG2 cells. Furthermore, the ginkgols with unsaturated side chains (C17:2, C15:1-Δ^8^, C17:1-Δ^12^, and C17:1-Δ^10^) exhibited more potent inhibitory effects than ginkgols with saturated side chains (C13:0, C15:0). In addition, unsaturated ginkgol C15:1-Δ^8^ showed the most potent cytotoxicity on HepG2 and HGC cells, of which the half-maximal inhibition concentrations (IC_50_) were 18.84 ± 2.58 and 13.15 ± 2.91 μM, respectively. The IC_50_ for HepG2 and HGC cells for the three unsaturated ginkgols (C17:1-Δ^10^, C17:2 and C17:1-Δ^12^) were ~59.97, ~60.82, and ~68.97 μM for HepG2 and ~30.97, ~33.81, and ~34.55 μM for HGC cells, respectively. Comparing the ginkgols’ structure–activity relations, the findings revealed that the position and number of the double bonds of the ginkgols with 17 side chain carbons in length had no significant difference in anticancer activity.

## 1. Introduction

**Figure 1:**
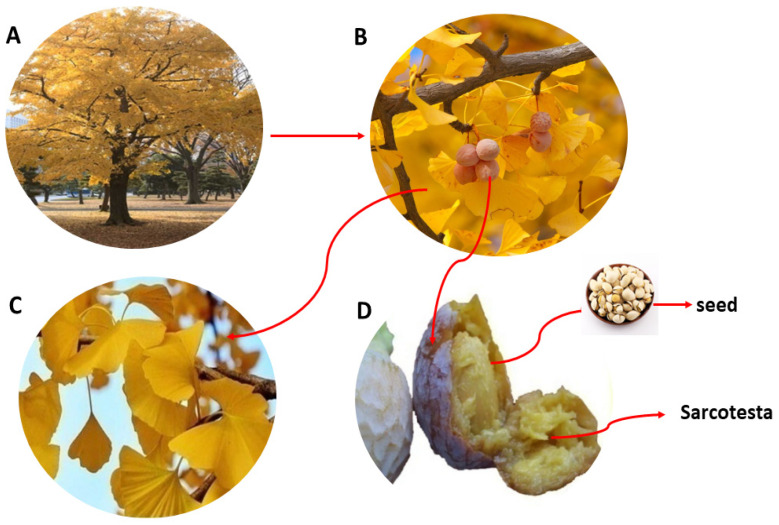
*Ginkgo biloba* tree and various parts: (**A**) tree, (**B**) fruit and leaves, (**C**) leaves, and (**D**) sarcotesta and seed.

One of the oldest surviving tree species on earth is the vast tree with fan-shaped leaves known as *Ginkgo biloba* (Figure 1A). It is referred to as a “living fossil” because it is the only remaining member of a long-extinct tree family that is 245 million years old [1]. Although it is native to China, Japan, and Korea, the GB tree has also been grown in temperate areas of Europe, United States, India, New Zealand, and Argentina [2]. Due to its abundance of bioactive compounds and diverse spectrum of pharmacological activities, GB is a widely utilized medicinal plant. Because of this, almost every part of GB (Figure 1A–D) has been the focus of in-depth study. Due to the bilobalide, ginkgolides, and flavonoids it possesses, *Ginkgo biloba* extract (GBE) is one of the most popular and extensively researched medicinal herbs. In addition, GBE has antioxidant, anticancer, anti-inflammatory, blood circulation-improving, and cardioprotective properties [3,4]. Thus, it is widely utilized in the functional food, cosmetics, and pharmacological industries, with sales of more than USD 10 billion since 2017 [5].

However, owing to the presence of ginkgolic acids (GAs) that are genotoxic [6,7], cytotoxic [7], allergenic [8], mutagenic, and neurotoxic [6], GBE’s usage has been restricted. In 2020, European, United States, and Chinese pharmacopoeias mandated that the GAs in GBE should be less than 5 µg/g [1]. Nevertheless, numerous pharmaceutically preferred impacts have been documented for using GAs, such as anticancer and antimicrobial activities [1]. The study of GA’s prospective uses as a medicinal agent for cancer treatment and various disorders is of interest as a result of these noteworthy bioactivities.

The GB sarcotestae and leaves (Figure 1C,D) contain large amounts of GAs, with the maximum concentrations being between 5% and 13% [9]. However, most GB sarcotestae are thrown away, damaging the environment and leading to resource wastage. Nonetheless, GAs can be extracted from the sarcotestae, which are then utilized as a biological insecticide [10]. Our previous research discovered that when GAs are heated, they will be converted into ginkgols (3-alkylphenols) by decarboxylation [11].

Ginkgols are the active compounds from GB and have pharmacological activities, such as antibacterial, antioxidant, and anti-apoptotic activities, on cancer cells in vitro [12,13]. Lepoittevin et al. reported that ginkgols failed to induce allergic contact dermatitis in an animal model. Meanwhile, ginkgolic acids seemed to be the main allergens of GB [14]. Therefore, ginkgol could be considered a potential drug with lower toxicity than GA. A previous study by Yang et al. [11] demonstrated that ginkgols exhibit superb thermostability and anticancer impacts as ginkgol C17:1, C15:1, and C13:0 were separated, and the effects of ginkgols on cancer cells were investigated. Nevertheless, in our findings from the GC-MS study, ginkgol C17:1 exhibited two peaks with similar mass spectra. Yang et al. [11] assumed that the two peaks represent isomers with various locations for the double bond. Thus, further research on the separation and identification of ginkgol C17:1 and evaluation of their bioactivities are needed.

Therefore, this research aimed to extract, purify, and elucidate six ginkgol homologs from GB sarcotesta for the first time and evaluate their anticancer activities. First, six ginkgol monomers are separated by HPLC. Then, the structures are identified by utilizing FTIR, UV, GC-MS, ^1^H-NMR, and ^13^C-NMR analyses. Aside from that, the positions of the double bonds are deduced by KMnO_4_ and NaIO_4_ oxidative decomposition, and their anticancer activities are juxtaposed using an MTT assay.

## 2. Results and Discussion

### 2.1. Preparation of Ginkgol

**Figure 2:**
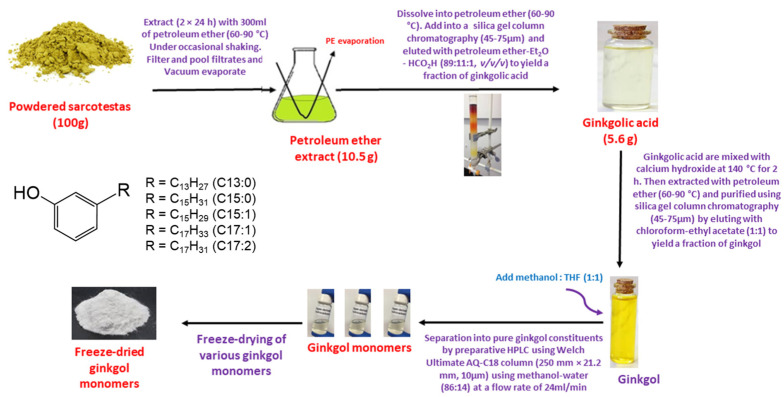
Schematic presentation of the ginkgols’ isolation.

**Figure 3:**
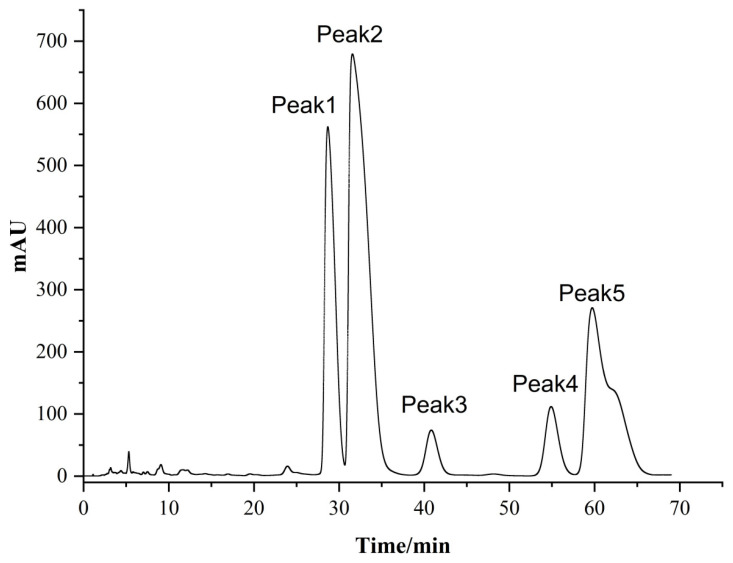
Preparative chromatogram of the ginkgols.

Ginkgolic acids (6-alkylsalicylic acids) and ginkgols (3-alkylphenols) occur in various parts of GB [15]. Both have similar structures, though the former only has one more carboxyl on the benzene ring than the latter. Of these two groups, ginkgolic acids were first noticed due to their allergenicity and higher concentrations than ginkols [16]. It was reported by van Beek et al. [16] that six GAs (C13:0, C15:0, C15:1, C17:1, C17:2, and C17:3) from *Ginkgo* leaves were separated based on a combination of reversed-phase mechanisms and double bond complexation by preparative chromatography. The separation processes were accomplished on the dual column of a C_18_ reversed-phase column connected in series with a silver ion-loaded cation exchanger column [16]. However, based on the complexation of double bonds and silver ions, the GA monomers with double bond isomers or geometric isomers are difficult to separate. Aside from that, restricted by the stability and toxicity of silver ions, this preparation method is not suitable for production of medicinal compounds. For the reasons above, establishing a simple one-pot HPLC method for separating the ginkgol monomers’ access to pure compounds is necessary. In this study, the entire isolation and purification procedure is schematically presented in Figure 2, which is divided into four steps: (1) GAs are extracted with petroleum ether, (2) isocratic separation is performed by silica gel column chromatography, (3) the GAs are transformed into ginkgols by thermal decarboxylation under alkaline condition, and (4) the ginkgol monomers are isolated by preparative RP-HPLC. In a second process, the purpose of initial crude extract isocratic separations was to concentrate the GAs by removing the co-extracted impurities. After repeated isocratic separations, more than 90% of the content of the GA fraction was obtained. The initial temperature of decarboxylation of GA is above 200 °C [11]. When Ca(OH)_2_ was mixed well with GA at a ratio of 0.02:1 (g/g), this decarboxylation could be completed in 2 h at 140 °C. In the final process, a one-pot RP-HPLC separation was carried out on 10-μm C_18_ column packing. The five principal ginkgol peaks were baseline separated (Figure 3) under optimal conditions. To obtain a pure compound, every peak was segmented according to the peak retention time and intensity according to the method described in Section 3.3, followed by fractional collection and pooling of the same fractions. As a result, six compounds (from G-1 to G-6) were obtained, with compounds G-5 and G-6 separated from the fifth peak for the first time. The analytical HPLC chromatograms showed the purity of the six compounds in Figure S4. Among the six compounds, G-1 and G-4 were white powder, and G-2, G-3, G-5, and G-6 were light yellow oil.

### 2.2. Identification of Ginkgols

**Figure 4:**
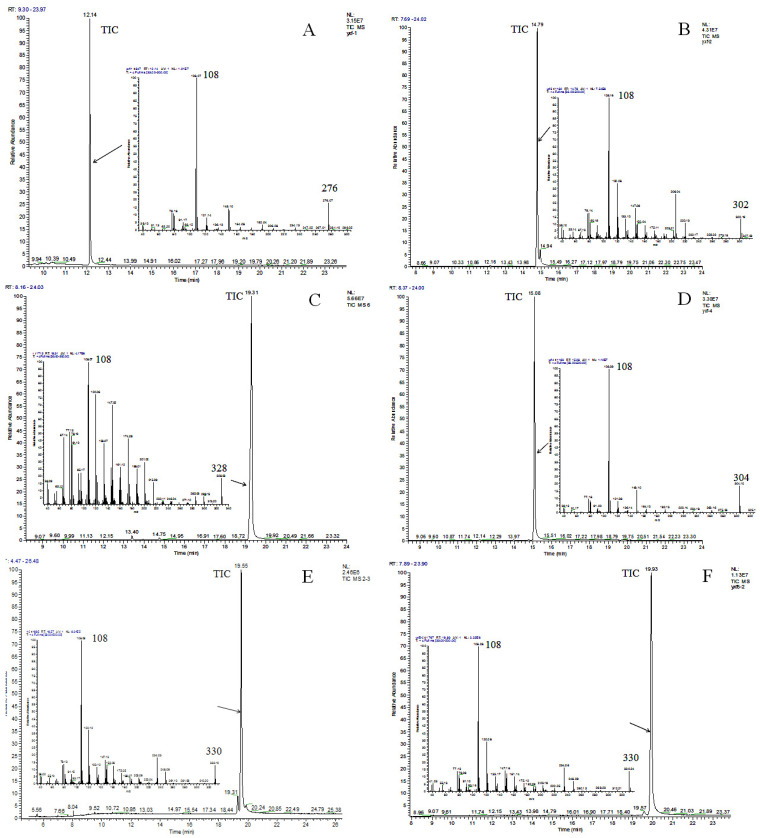
The GC-MS chromatogram and EI mass of compounds G-1 (**A**), G-2 (**B**), G-3 (**C**), G-4 (**D**), G-5 (**E**), and G-6 (**F**).

The length of the capillary column of the GC was 30 m, while that of the LC column was only 250 mm. Thus, the isomers were better separated on the GC capillary column than on the LC column [16]. GC-MS further identified the purity of these compounds (Figure 4). The total ion chromatogram (TIC) of G-1 is shown in Figure 4A, which appeared as a symmetrical and single peak. The TICs of G-2, G-3, G-4, G-5, and G-6 are shown in Figure 4B–F, and all had a symmetrical and single peak. The area normalization method [17] was adopted to calculate the purity of the 6 ginkgols, which were found to be 100%, 98.1%, 100%, 100%, 96.7%, and 99% for G-1–G-6, respectively.

The six ginkgols’ structures were identified by FTIR, UV, ^13^C-NMR, and ^1^H-NMR spectra and GC-MS (Figure 4 and Table 1). The spectra data are shown in Table 2. The ^1^H-NMR data for the six ginkgols demonstrated the presence of a methyl group, extended chain methylene groups (δ_H_ ~1.3 ppm, br) with an δ_H_ of approximately 0.9 ppm (3H, t, *J* = ~6.8 Hz), and 1, 3-substituted benzene (δ_H_ of 7.15 (1H, t, *J* = ~7.6 Hz,), 6.76 (1H, d, *J* = ~7.6 Hz), and 6.66 (2H, m) ppm). FTIR spectra (Table 2 and Figure S1) (phenolic hydroxyl stretching vibration (ν_O-H_) at a wavenumber of ~3330 cm^−1^) [18,19] were used to demonstrate the presence of hydroxyl groups. The *m*/*z* 91 (C7H7^+^, tropylium ion) indicated the occurrence of alkyl substitution on the benzene ring. The six ginkgols’ UV spectra (λ_max(MeOH)_ = 275 nm) are shown in Table 2 and Figure S2. The above data show that they all were 3-alkyl phenol [20,21]. However, the six ginkgols differed in terms of the number of double bonds or the length of the alkyl chain.

The alkyl chains’ lengths and unsaturation were determined by the ^13^C-NMR, ^1^H-NMR, and molecular ion peaks in the MS. As shown in Figure 4A–F, the molecular ion peaks from G-1 to G-6 (*m*/*z* 276, 302, 328, 304, 330, and 330, respectively) corresponded to ginkgols with the alkyl chains C13:0, C15:1, C17:2, C15:0, C17:1, and C17:1, respectively. Since it was anticipated that the two compounds correlated with isomers with the double bond in different places or as cis-trans isomerisms [11], the compounds G-5 and G-6 showed identical mass spectra.

**Figure 5:**
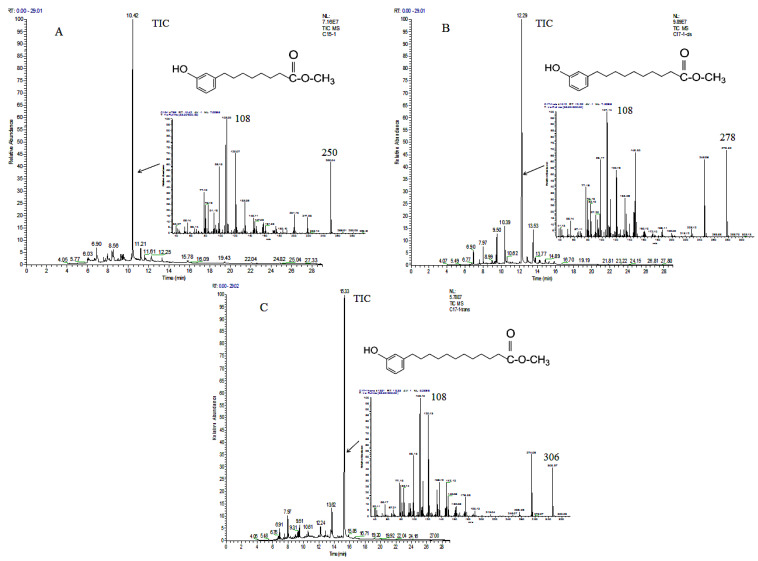
The GC-MS chromatogram and EI mass of the methyl esters of the compounds G-2 (**A**), G-5 (**B**), and G-6 (**C**).

**Figure 6:**
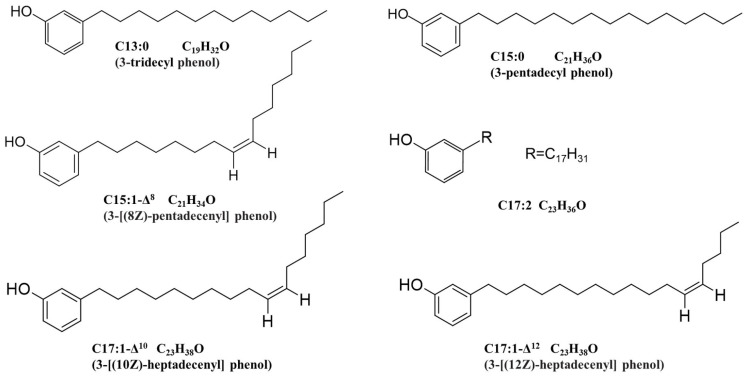
Structures of the ginkgol monomers.

To completely characterize the precise positions of the G-2 (C15:1), G-5 (C17:1), and G-6 (C17:1) double bonds, they were degraded oxidatively with KMnO_4_ and NaIO_4,_ and the produced acids were methylated utilizing (CH_3_OH)_2_ BF_3_, and their methyl esters products (MEPs) were examined by GC-MS (Figure 5). The molecular ion peak of the methyl esters of the oxidation products was used to determine the double bonds’ positions in the alkyl chain [20]. For instance, the NMR signal for G-5 at δ_H_ 5.36 (2H) ppm, δ_C_ 129.9 (2C) ppm demonstrated a double bond in the alkyl chain. The geometric structure of the double bond in C17:1 is cis or trans, which can be judged by the coupling constant. For δ_H_ 5.36 ppm (2H, m, *J* = 4.8 Hz, CH=CH), the cis coupling constants usually lies in the range of 3–13 Hz [22]. Therefore, the double bond was determined to be cis, and its position was Δ^10^, combined with the molecular ion peak (*m*/*z* 278) in the MS of the MEP (Figure 5B). Compound G-5 was identified as 3-[(10Z)-heptadecenyl] phenol (Figure 6). At the same time, G-2 and G-6’s double bonds were established to be Δ8-C15:1 (δ_H_ 5.37, *J* = ~2.0 Hz, 2H, CH=CH) and Δ^12^-C17:1 (δ_H_ 5.36, *J* = ~4.4 Hz, 2H, CH=CH) based on their molecular ion peaks (*m*/*z* 250 and 306, respectively) in the MS of their MEPs (Figure 5A,C). Thus, G-2 was labeled as 3-[(8Z)-pentadecenyl] phenol (Figure 6), and G-6 was labeled as 3-[(12Z)-heptadecenyl] phenol (Figure 6) [20,23,24]. The G-1 and G-4 compounds′ spectra data were consistent with earlier studies [20,21,23,24,25]. Therefore, these compounds were labeled as 3-tridecyl phenol and 3-pentadecyl phenol, respectively. Their structural formulae are enumerated in Figure 6. However, as ginkgol C17:2 was not subjected to oxidative degradation experiments, its double bond position could not be determined. According to the reports of Irie et al. [20], van Beek et al. [16], and Yang et al. [11] on ^1^HNMR and ^13^CNMR, the positions of C and H were assigned, and the results are shown in Table 2. The ^1^HNMR and ^13^CNMR spectra are shown in Figure S3.

### 2.3. Anticancer Activity of Ginkgol Monomers

**Figure 7:**
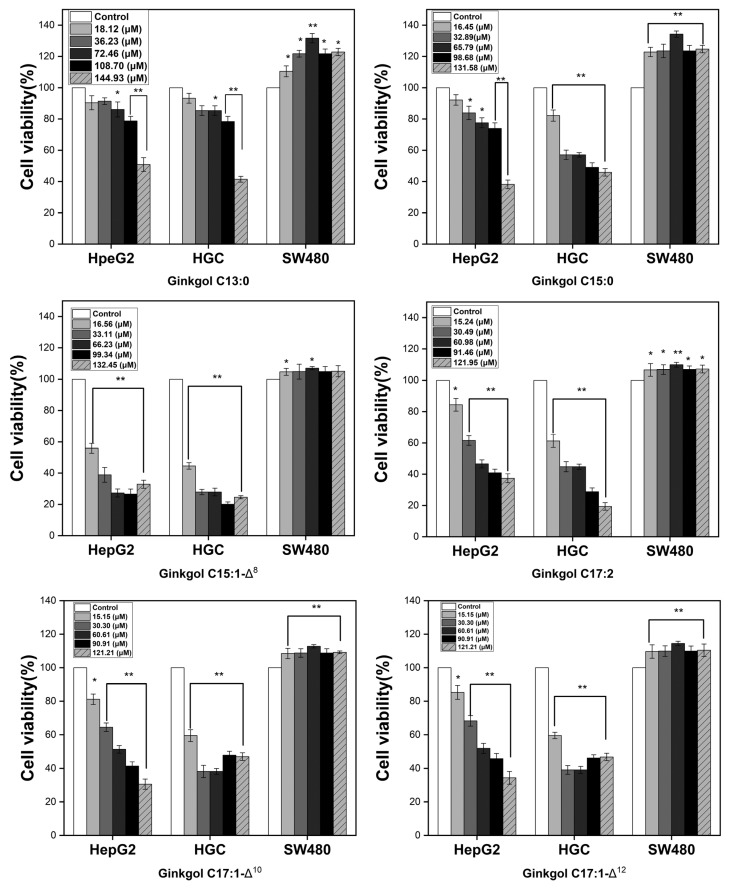
Inhibitive effects of ginkgol C13:0, C15:1-Δ^8^, C17:2, C15:0, C17:1-Δ^10^, and C17:1-Δ^12^ on HepG2, HGC, and SW480 cell lines at different concentrations. * *p* < 0.05 vs. control. ** *p* < 0.01 vs. control.

The MTT method was used to assess how these six ginkgol monomers inhibited the growth of the malignant HepG2, HGC, and SW480 cells. Six monomers were applied in escalating doses to all cells tested for 24 h (Figure 7). The results showed that the SW480 cells displayed a growth-promoting impact with negative inhibition rates when exposed to six ginkgol monomers. Qiao et al. [26] reported that the inhibitory effect of ginkgolic acid treatment on the proliferation of SW480 cells was concentration- and time-dependent, where the higher the concentration of ginkgolic acid, the greater the degree of inhibition. However, ginkgol showed the opposite result against the SW480 cells in our test. This may be because ginkgol monomers lack the carboxyl group compared with ginkgolic acid and lose the cytotoxicity to SW480 cells. As Ji et al. [27] pointed out, phenolic acids, which possess a carboxyl group, are more active than the corresponding cardanols (ginkgol). However, all six ginkgol monomers had significant growth inhibition on HepG2 and HGC cells (Figure 7). It was observed that the sensitivity of various cancer cell lines to each of these bioactive compounds varied significantly (*p* < 0.05). The six ginkgol monomers’ IC_50_ against HepG2 and HGC cells were computed and are shown in Table 3. Compared with the HepG2 cell, the HGC cell was more responsive to all ginkgols with lower IC_50_ values (Table 3). When tested against HepG2 and HGC cells, the ginkgols with unsaturated alkyl sidechains were more lethal than those with saturated side chains (IC_50_ values increasing in the order of C13:0 > C15:0 > C17:1-Δ^12^ > C17:1-Δ^10^ > C17:2 > C15:1-Δ^8^). This conclusion is similar to the results of Kim et al. [28], who pointed out that the double bond in the side chain of alkylphenol may add to cytotoxicity by causing steric hindrance in membrane penetration. Ji et al. [27] also pointed out that unsaturated long-chain substituents may be vital for PI-PLCγ1 inhibitory activity.

In the 4 ginkgol monomers with unsaturated sidechains (C15:1-Δ^8^, C17:1-Δ^10^, C17, and C17:1-Δ^12^), ginkgol C15:1-Δ^8^ showed the most potent cytotoxicity on HepG2 and HGC cells, of which the 24h-IC_50_ values were 18.84 ± 2.58 and 13.15 ± 2.91 μM, respectively (Table 3). The other three ginkgol monomers were C17:1-Δ^10^, C17:2, and C17:1-Δ^12^, with sidechain lengths of 17 carbons and 1–2 double bonds in a different position (Table 3). Their 24 h-IC_50_ values against HepG2 and HGC cells were ~59.97 and ~30.97 μM, ~60.82 and 33.81 μM, and ~68.97 and 34.55 μM, respectively. The results show that the toxicity effects decreased in the order of C17:1-Δ^10^, C17:2, and C17:1-Δ^12^ but with no significant difference (*p* > 0.05). Ji et al. [27] reported that they only isolated five ginkgol monomers (C13:0, C15:1-Δ^8^, C17:1-Δ^10^, C15:0, and m-Cresol C1:0) from the sarcotestas of GB. For our study, six ginkgol monomers were efficiently separated. In particular, two isomers of ginkgol C17:1 (C17:1-Δ^10^ and C17:1-Δ^12^) and ginkgol C17:2 were obtained, so the influence of the position and the number of double bonds on anticancer ability could be explored. According to our experimental data, the position and the number of double bonds in the side chain had no noticeable effect on the anticancer activity of ginkgol with a side chain 17 carbons in length. In addition, ginkgol C15:1 showed more potent cytotoxicity to HepG2 and HGC cells than ginkgol C17:1 (Table 3), which was contrary to our previous study [11]. Our earlier research [11] indicated that a ginkgol C17:1 (mixture made up of 2 isomers at a ratio of 1:1) had more potent inhibitory effects on SMMC7721, U251, and A549 cells than ginkgol C15:1. Li et al. [13] also reported that among the ginkgol monomers, ginkgol C17:1 has been shown to exert the strongest inhibitory effect on cell viability in a number of human cancer cells, which inhibit HepG2 cell growth by blunting the EGF-PI3K/Akt signaling pathway. As far as we know, Yang et al. [11], Li et al. [13,29] and Liu et al. [30] all used a mixture of two isomers of ginkgol C17:1. Therefore, we supposed that the two isomers of ginkgol C17:1 (C17:1-Δ^12^ and C17:1-Δ^10^) would have a synergistic effect on inhibiting HepG2 cell growth. Their cytotoxicity was reduced when presented in monomeric form. Another probability was that a compound with higher lipophilicity could more easily penetrate the cell membrane and show higher cytotoxicity. However, chain lengths exceeding certain limits may attenuate cytotoxicity, possibly because of the limited solubility in the aqueous phase, which leads to the compounds being “trapped” in the outer cellular membrane [6]. Thus, C15:1-Δ^8^ has a double bond and a suitable side chain length which exhibits superior anticancer activity compared with ginkgol C17:2, C17:1-Δ^12^, and C17:1-Δ^10^.

Li et al. [29] reported that the ginkgol C17:1 mixture inhibited the growth of HepG2 cells, with a 24h-IC_50_ value of ~484.85 μM, of which the difference was eight times that of our results (24h-IC_50_ values of ~59.97 μM and ~68.97 μM) (Table 3). In another of their papers, the 24h-IC_50_ value of the ginkgol C17:1 mixture was ~121.21 μΜ [13], which was close to our result. The differences between their two experimental results may be related to the preparation of the ginkgol stock and working solution with DMSO. When the concentration of ginkgol in the culture medium is ~484.85 μM, the concentration of its stock solution should not be lower than 48.49 mM to ensure that the DMSO concentration is lower than 0.1% when mixed with the cell suspension in a 96-well plate. This is a great challenge to the solubility of DMSO. In the case of incomplete dissolution, the ginkgo is not uniformly dispersed in DMSO, which increases the experimental error. Therefore, the results of their two experiments (24-h IC_50_ values of ~484.85 μM and ~121.21 μM) are different and higher than ours (24-h IC_50_ values of ~59.97 μM and ~68.97 μM). Cisplatin is one of the most useful anticancer agents available for cancer therapy [31]. Li et al. [30] reported that the combination of ginkgol C17:1 with a low dose of cisplatin (6.67 μM) exhibited prominent cytotoxicity in hepatoma cells. When cisplatin alone inhibits HepG2, its 24h-IC_50_ is about 53.32 μM. This is close to but lower than the IC_50_ value of the ginkgol C17:1 isomers measured in our study and about 2.8 times higher than the IC_50_ value of ginkgol C15:1. Ginkgol C15:1 may offer outstanding performance in terms of anticancer properties compared with established drugs, but further research is needed.

## 3. Materials and Methods

### 3.1. Materials and Chemicals

Sunlight-dried GB sarcotestas were collected in October at Zhenjiang city (Zhenjiang, China) and authenticated at Jiangsu University’s School of Pharmaceuticals (Pharmacognosy Laboratory), where the voucher specimens were stored. The dried GB sarcotestas were ground into powder, sieved (40−60 mesh), and stored in a dryer (27 °C). Methanol (HPLC grade) was bought from TEDIA (Shanghai, China). The HPLC tests were conducted with purified water (Wahaha Co., Ltd., Hangzhou, China). Unless otherwise stated, all additional chemicals and reagents utilized in this investigation were of analytical grade and bought from Sinopharm Co., Ltd. (Shanghai, China). The fetal bovine serum and Dulbecco’s modified eagle medium (DMEM) were bought from Gibco (Shanghai, China). The (CH_3_OH)_2_BF_3_-14% and MTT were bought from Sigma-Aldrich (St. Louis, MO, USA). HepG2 (hepatocellular carcinoma), SW480 (colon cancer), and human gastric cancer (HGC) cancer cell lines were bought from the Institute of Cell Biology and Biochemistry (Shanghai, China). The cells were acclimated (37 °C) in a humidified atmosphere (5% of CO_2_) in DMEM media containing 1% mixed solution of streptomycins (10 mg/mL) and penicillin (10 kU/mL) and 10% fetal bovine serum.

### 3.2. Extraction and Decarboxylation of Gingkolic Acids

The complete isolation and purification process is shown schematically in Figure 2. First, isolation of GA from the sarcotesta followed our previous protocol [11]. The dried GB sarcotesta was extracted to yield petroleum ether extracts (Figure 2), which were then separated by column chromatography (silica gel) and eluted using Et_2_O, petroleum ether, and HCO_2_H (11:89:1, *v*/*v*/*v*) to yield a GA fraction. The separation process was repeated twice, and the GA homologs mixture (purity > 90%) was obtained. Next, the GA’s decarboxylation process was carried out as described by Paramashivappa et al. [32]. Ca(OH)_2_ was added to the GAs at a ratio of 0.02:1 (g/g) and heated at 135~140 °C for 2 h. When the mixture was cooled to room temperature, the mixture was extracted with petroleum ether (boiling range of 60~90 °C), filtered, and concentrated to obtain a brown oil which was further purified by column chromatography (silica gel) to obtain the ginkgol homolog mixture, as shown in Figure 2.

### 3.3. Isolation and Purification of Ginkgol Monomers

Isolation and purification of the ginkgol monomers was based on the protocol of Yang et al. [11] with some modifications. The ginkgols’ separations were carried out using a preparative HPLC which consisted of two WK500LC-500P pumps coupled to a UV detector (SPD-500) and an injector (Marathon XT) with a 1-mL loop (Xu Yu Technology Co., Ltd., Hangzhou, China). The AQ-C18 column (250 mm × 21.2 mm, 10 μm, Welch Ultimate, Shanghai, China) was used for the separation, and a UV detector was used to monitor the ginkgol monomers at 280 nm. The mobile phase used for the elution was MeOH and H_2_O (86:14, *v*/*v*) at a flow rate of 24 mL/min.

Following the isolation method mentioned and shown in Figure 2, the five principal ginkgol peaks were baseline separated (Figure 3) under optimal conditions, and the separation procedure took 68 min to complete. To obtain a pure compound, every peak was segmented according to the peak retention time and intensity. For the first peak, three fractions were collected according to the retention time at 28.1–28.6 min, 28.6–29.7 min, and 29.7–30.2 min. For the second peak, four fractions were collected from the eluent at 30.3–30.7 min, 30.7–32.0 min, 32.0–33.8 min, and 33.8–35.5 min. For the third peak, three fractions were collected at 39.1~39.6 min, 39.6~41.9 min, and 41.9~42.8 min. For the fourth peak, three fractions were collected at 54.1~54.6 min, 54.6~55.9 min, and 55.9~56.5 min. In Figure 3, in front of these four peaks, the symmetrical spikes are shown. In contrast, the fifth peak looked like a shoulder peak, which indicates that it could contain more than one compound. Hence, we divided it into 7 parts in the order of 58.5~59.0 min, 59.0~60.7 min, 60.7~62.3 min, 62.3~63.4 min, 63.4~64.3 min, 64.3~66 min, and 66~67.5 min. After that, all the fractions were examined with the HPLC protocol for analysis, and the fractions that displayed the same peak shape and retention time were combined.

Regarding the purification of ginkgol mononers, an HPLC system (Agilent 1260, Agilent Corp., Santa Clara, CA, USA) coupled to a C18 column (250 mm × 4.6 mm, 5 μm, a Shimp-pack VP-ODS, Shimadzu Co., Kyoto, Japan) was used. Methanol and water (90:10, *v*/*v*) were utilized as the mobile phase at a flow rate of 1 mL/min. At a wavelength of 275 nm, the ginkgols in the eluent were detected. Data collection and processing were carried out using Agilent Chemstation software B.02.

### 3.4. Structural Characterization of Ginkgol Monomers

#### 3.4.1. Ultraviolet (UV), Fourier Transform Infrared (FTIR), and NMR Spectra

The UV spectra for the ginkgol monomers were obtained using methanol as the solvent [11] on a UV spectrometer (UV-2450, Shimadzu Co., Ltd., Japan). The FT-IR spectra for the ginkgol monomers were recorded on an FT-IR spectrometer (Nicolet Nexus 470, Thermo Scientific, Rockford, IL, USA) using dichloromethane as the diluting agent [11].

In terms of the NMR analysis, the procedure of Yang et al. [11] was utilized. Tetramethylsilane (TMS) was used as an internal standard while collecting NMR spectra on an NMR spectrometer (DRX500, Bruker, MA, USA) in deuterated chloroform at 100 and 400 MHz for ^13^C and ^1^H analysis, respectively.

#### 3.4.2. GC-MS Analysis

GC-MS analysis was accomplished based on the protocol of Yang et al. [11] with minor modifications. First, a GC system (Ultra TRACE Thermo Fisher Scientific, Waltham, MA, USA) coupled to a selective detector (ITQ1100) which was equipped with a capillary column (0.25 µm, 0.25 mm, 30 m length, TR-5MS, Thermo Fisher Scientific, MA, USA) was applied.

The carrier gas (helium, 1 mL/min) had a linear pressure (0.38 MPa). A 1-μL sample of aliquots was introduced into the GC apparatus at a split ratio of 1:20. In the analytical settings, the initial temperature was 100 °C (2 min), increased to 240 °C at 20 °C/min, and held constant at 240 °C for 20 min. The spectra were collected using an electron impact ion source (EI) between 25 and 550 Da.

#### 3.4.3. Assessment of Ginkgols’ Olefinic Chains

Adopting the technique proposed by Irie et al. [20], the locations of the double bonds were assessed using oxidative degradation. First, the ginkgol monomer (1 mg) was dissolved in ter-BuOH (1 mL) before being combined with 1 mL of KMnO_4_ (5 mM)/NaIO_4_ (20 mM) and Na_2_CO_3_ (1 mL, 4 mM) and stirred (1 h, 27 °C). Then, it was extracted with 10 mL of *n*-hexane three times and left to evaporate until dry. Third, (CH_3_OH)_2_BF_3_-14% was used to methylate the generated acid. Finally, the methyl esters products (MEPs) were located using GC-MS as described in Section 3.4.2 to determine the location of the final double bond in the chain.

### 3.5. Anticancer Activity Assay

The cytotoxicity of the ginkgols was determined using an MTT assay based on the procedure of Qiao et al. [26] with some modifications. The ginkgol was dissolved with DMSO to prepare a stock solution of 18 mM and then diluted with DMSO into a series of concentrations of work solutions. The cells were pooled and diluted to a cell density of 5 × 10^4^ cells/mL, and then a 100-μL cell suspension was seeded into each well of a 96-well plate. Following incubation in DMEM supplemented with 10% FBS for 24 h at 37 °C in 5% CO_2_, the cells were treated with various concentrations (the corresponding molarities (μM) are shown in Table S1) of ginkgol monomers in the same final volume of 100 μL per well (DMSO content of 0.1%) for an extended incubation period of 24 h. The control (ginkgol-free) group received the same treatment time with a culture medium containing 0.1% DMSO, after which both were exposed to MTT (5 mg/mL, 20 μL) for an additional 4 h. Subsequent to removal of the growth medium, 100 µL of dimethyl sulfoxide (DMSO) was added each well and allowed to oscillate completely to dissolve the resulting formazan crystals. The absorbance was measured at 570 nm using a microplate reader (iMark, Bio-Rad, Hercules, CA, USA). Each group was provided with six parallel holes. The cell viability (%) and inhibition rate (%) were calculated using the formulae below:

$$\mathrm{Cell}\;\mathrm{viability}\;\left(\%\right)=\frac{A_{\mathrm{sample}}}{A_{\mathrm{control}}}\times 100\%$$

$$\mathrm{Inhibition}\;\mathrm{rate}\;\left(\%\right)=\frac{A_{\mathrm{control}}-A_{\mathrm{sample}}}{A_{\mathrm{control}}}\times 100\%$$

### 3.6. Statistical Analysis

The data were displayed as the mean ± standard deviation. One-way analysis of variance (ANOVA) using Minitab version 2021 was used for analysis (Minitab Inc., State College, PA, USA). Tukey’s test was also used to juxtapose the means at *p* < 0.05. Graphical representations were performed using Origin 2021 software (Origin Lab Inc., Northampton, MA, USA). The IC_50_ value was calculated based on IBM SPSS Statistics 26.0 (New York, NY, USA). The experiments were carried out in triplicate.

## 4. Conclusions

Six pure ginkgol monomers were separated and identified from ginkgol homologs using a simple one-pot separation method by RP-HPLC. First, the ginkgol C17:1 mixture was entirely separated into the two ginkgol C17:1 isomers (C17:1-Δ^12^ and C17:1-Δ^10^). Then, their anticancer activities were tested by the MTT method. The findings and analysis of the structure–activity relationship demonstrated that the ginkgol monomers with unsaturated side chains showed more potent activities than the saturated side chains. C15:1 in six ginkgols had the most potent inhibitory impact on cancer HepG2 and HGC cells, and it can be further studied as an excellent anticancer drug. Nonetheless, the difference in IC_50_ values of ginkgol C17:1-Δ^10^, C17:2, and C17:1-Δ^12^ was statistically insignificant (*p* > 0.05), indicating that the number and positions of the double bonds of unsaturated ginkgol with 17 sidechain carbons in length had no significant differences in antitumor activity. The low content of ginkgolic acid C17:2 in *Ginkgo biloba* sarcotesta makes the preparation of ginkgol C17:2 difficult, but the double bond position of ginkgol C17:2 needs to be further identified. Since the two ginkgol isomers (C17:1-Δ^12^ and C17:1-Δ^10^) were confirmed and identified for the first time, the anticancer mechanism of cancer cells needs to be further explored. Aside from that, future research needs to be conducted on whether the two ginkgol isomers affect cancer cells using in vivo studies.

## Figures and Tables

**Table 1:** EI mass fragments of isolated ginkgol monomers.

Peak	Retention Time (min)	Compound	*m*/*z*							
G-1	12.14	C13:0	276 (M^+^)	175	147	133		108 (100%)	91	77
G-2	14.79	C15:1-Δ^8^	302 (M^+^)	206	175	147	133	108 (100%)	91	77
G-3	19.31	C17:2	328 (M^+^)	232	175	147	133	108 (100%)	91	77
G-4	15.08	C15:0	304 (M^+^)	175	147	133		108 (100%)	91	77
G-5	19.55	C17:1-Δ^10^	330 (M^+^)	234	175	147	133	108 (100%)	91	77
G-6	19.93	C17:1-Δ^12^	330 (M^+^)	234	175	147	133	108 (100%)	91	77

**Table 2:** ^1^H-NMR, ^13^C-NMR, UV, and FTIR spectra of isolated ginkgol monomers.

**Ginkgol**	**Spectrum**	**Aryl**
C13:0	^1^H ^a^	6.67 (m, 2H, H2′, H4′), 6.76 (d, 1H, H6′), 7.15 (t, 1H, H5′)
	^13^C ^a^	112.5 (C6′), 115.3 (C2′), 120.7 (C4′), 129.3 (C5′), 144.9 (C3′), 155.5 (C1′)
	UV-(MeOH)	λ_max_ 275 nm
	FTIR (cm^−1^)	3353 (ν_O-H_), 1593 (ν_C=C_), 1458 (ν_C=C_), 1265 (ν_C-O_), 1154 (δ_=C-H_), 778 (δ_=C-H_), 695 (δ_C-O-H_)
C15:1-Δ^8^	^1^H	6.66 (m, 2H, H2′, H4′), 6.77 (d, 1H, H6′), 7.15 (t, 1H, H5′)
	^13^C	112.5 (C6′), 115.3 (C2′), 120.7 (C4′), 129.3 (C5′), 144.9 (C3′), 155.5 (C1′)
	UV-(MeOH)	λ_max_ 275 nm
	FTIR (cm^−1^)	3360 (ν_O-H_), 1593 (ν_C=C_), 1457 (ν_C=C_), 1265 (ν_C-O_), 1154 (δ_=C-H_), 778 (δ_=C-H_), 694 (δ_C-O-H_)
C17:2	^1^H	6.66 (m, 2H, H2′, H4′), 6.76 (d, 1H, H6′), 7.15 (t, 1H, H5′)
	^13^C	112.3 (C6′), 115.3 (C2′), 121.0, 129.4 (C5′), 144.9 (C3′), 155.5 (C1′)
	UV-(MeOH)	λ_max_ 275 nm
	FTIR (cm^−1^)	3338 (ν_O-H_), 1581 (ν_C=C_), 1456 (ν_C=C_), 1406 (δ_O-H_), 1265 (ν_C-O_), 1154 (δ_=C-H_), 778 (δ_=C-H_), 694 (δ_C-O-H_)
C15:0	^1^H	6.68 (m, 2H, H2′, H4′), 6.77 (d, 1H, H6′), 7.15 (t, 1H, H5′)
	^13^C	112.5 (C6′), 115.3 (C2′), 120.8 (C4′), 129.3 (C5′), 144.9 (C3′), 155.5 (C1′)
	UV-(MeOH)	λ_max_ 275 nm
	FTIR (cm^−1^)	3328 (ν_O-H_), 1593 (ν_C=C_), 1458 (ν_C=C_), 1265 (ν_C-O_), 1154 (δ_=C-H_), 777 (δ_=C-H_), 695 (δ_C-O-H_)
C17:1-Δ^10^	^1^H	6.65 (m, 2H, H2′, H4′), 6.76 (d, 1H, H6′), 7.15 (t, 1H, H5′)
	^13^C	112.4 (C6′), 115.3 (C2′), 120.9 (C4′), 129.3 (C5′), 144.9 (C3′), 155.4 (C1′)
	UV-(MeOH)	λ_max_ 275 nm
	FTIR (cm^−1^)	3307 (ν_O-H_), 1596 (ν_C=C_), 1456 (ν_C=C_), 1266 (ν_C-O_), 1153 (δ_=C-H_), 738 (δ_=C-H_), 697 (δ_C-O-H_)
C17:1-Δ^12^	^1^H	6.65 (m, 2H, H2′, H4′), 6.76 (d, 1H, H6′), 7.15 (t, 1H, H5′)
	^13^C	112.5 (C6′), 115.3 (C2′), 120.9 (C4′), 129.3 (C5′), 145 (C3′), 155.4 (C1′)
	UV-(MeOH)	λ_max_ 275 nm
	FTIR (cm^−1^)	3306 (ν_O-H_), 1594 (ν_C=C_), 1458 (ν_C=C_), 1266 (ν_C-O_), 1154 (δ_=C-H_), 738 (δ_=C-H_), 697 (δ_C-O-H_)
**Ginkgol**	**Spectrum**	**Side Chain**
C13:0	^1^H ^a^	0.9 (t, 3H, H13′), 1.29 (m, 20H, H3′–12′), 1.60 (m, 2H, H2′), 2.56 (t, 2H, H1′)
	^13^C ^a^	14.1 (C13′), 22.7 (C12′), 29.3, 29.5, 29.7, 30.9, 31.3 (C2′), 31.9 (C11′), 35.8 (C1′)
	FTIR (cm^−1^)	2925 (ν_C-H_), 2855 (ν_C-H_)
C15:1-Δ^8^	^1^H	0.90 (t, 3H, H15′),1.31 (m, 16H, H3′–6′, H11′–14′), 1.61 (m, 2H, H2′), 2.02 (m, 4H, H7′, H10′), 2.57 (t, 2H, H1′), 5.35 (m, 2H, CH=CH, H8′, H9′)
	^13^C	14.1 (C15′), 22.7 (C14′), 27.2 (C8′), 27.2 (C9′), 29.0, 29.2, 29.3, 29.4, 29.7, 31.3 (C2′), 31.8 (C13′), 35.8 (C1′), 129.6 (C9′), 130.0 (C8′)
	FTIR (cm^−1^)	2926 (ν_C-H_), 2855 (ν_C-H_)
C17:2	^1^H	0.90 (t, 3H, H17′), 1.32 (m, 14H, H3′–7′, H15′–16′), 1.60 (m, 2H, H2′), 2.06 (m, 4H, H8′, H14′), 2.56 (t, 2H, H1′), 2.78 (t, 2H, =CH-CH_2_-CH=, H14′), 5.37 (m, 4H, CH=CH, H9′–10′, H12′–13′)
	^13^C	14.1 (C17′), 22.6 (C16′), 25.7, 27.2, 29.2, 29.3 (2C), 29.4, 29.7, 29.7, 31.3 (C2′), 31.6 (C15′), 35.8 (C1′), 128.0, 128.0, 130.1, 130.2
	FTIR (cm^−1^)	2925 (ν_C-H_), 2855 (ν_C-H_)
C15:0	^1^H	0.91 (t, 3H, H15′), 1.30 (m, 24H, H3′–14′), 1.61 (m, 2H, H2′), 2.57 (t, 2H, H1′)
	^13^C	14.1 (C15′), 22.7 (C14′), 29.3, 29.3, 29.5, 29.6, 29.7, 30.9, 31.3, 31.9 (C13′), 35.8 (C1′)
	FTIR (cm^−1^)	2925 (ν_C-H_), 2855 (ν_C-H_)
C17:1-Δ^10^	^1^H	0.89 (t, 3H, H17′), 1.29 (m, 20H, H3′–8′,H13′–16′), 1.60 (m, 2H, H2′), 2.02 (m, 4H, H8′, H9′), 2.56 (t, 2H, H1′), 5.36 (m, 2H,CH=CH, H10′, CH11′)
	^13^C	14.1 (C17′), 22.7 (C16′), 27.2 (C9′), 27.2 (C12′), 29.0, 29.3, 29.5, 29.5, 29.7, 29.7, 29.8, 31.3 (C2′), 31.8 (C15′), 35.8 (C1′), 129.9 (C10′), 129.9 (C11′)
	FTIR (cm^−1^)	2925 (ν_C-H_), 2855 (ν_C-H_)
C17:1-Δ^12^	^1^H	0.91 (t, 3H, H17′), 1.31 (m, 20H, H3′–H10′, H15′–16′), 1.60 (m, 2HC2′), 2.02 (m, 4H, H11′, H14′), 2.56 (t, 2H, H1′), 5.36 (m, 2H, CH=CH, H12′, H13′)
	^13^C	14.0 (C17′), 22.3 (C16′), 26.9 (C14′), 27.2 (C11′), 29.3, 29.5, 29.5, 29.6, 29.6, 29.6, 29.7, 29.8, 31.3 (C2′), 32.0 (C15′), 35.8 (C1′), 129.8 (C13′), 129.9 (C12′)
	FTIR (cm^−1^)	2925 (ν_C-H_), 2855 (ν_C-H_)

Note: ^a^ NMR values in ppm relative to TMS. UV = ultraviolet; FTIR = Fourier transform infrared.

**Table 3:** Inhibition effects of the ginkgol compounds on the HepG2 and HGC cells.

Cell Lines	24 h-IC_50_ (μM)					
	C13:0	C15:1-Δ^8^	C17:2	C15:0	C17:1-Δ^10^	C17:1-Δ^12^
HepG2	156.81 ± 3.01 ^Bd^	18.84 ± 2.58 ^Ba^	60.82 ± 2.90 ^Bb^	128.09 ± 2.60 ^Bc^	59.97 ± 3.01 ^Bb^	68.97 ± 3.00 ^Bb^
HGC	137.31 ± 3.22 ^Ad^	13.15 ± 2.91 ^Aa^	33.81 ± 2.74 ^Ab^	97.80 ± 3.22 ^Ac^	30.97 ± 1.03 ^Ab^	34.55 ± 1.45 ^Ab^

Note: Different capital letters in the same column represent significant differences (*p* < 0.05). Different lowercase letters in the same row represent significant differences (*p* < 0.05).

## Data Availability

Not applicable.

## Supplementary Materials

The following supporting information can be downloaded at: https://www.mdpi.com/article/10.3390/molecules27227777/s1, Figure S1: FTIR spectra of six ginkgols, Figure S2: UV spectra of six ginkgols, Figure S3: ^1^HNMR, and ^13^CNMR spectra of six ginkgols, Figure S4: The chromatograms of the various ginkgols G-1, G-2, G-3, G-4, G-5, and G-6. Table S1: Molarity of six ginkgols for anticancer activity assay.

## Abbreviations

GB	*Ginkgo biloba*
GA	Ginkgolic acid
GBE	*Ginko biloba* extract
HPLC	High-performance liquid chromatography
RP-HPLC	Reversed-phase high-performance liquid chromatography
UV	Ultraviolet
FTIR	Fourier transform infrared
GC-MS	Gas chromatograph mass spectrometer
^1^H-NMR	Proton nuclear magnetic resonance
^13^C-NMR	Carbon-13 nuclear magnetic resonance
IC_50_	Half-maximal inhibition concentration
MTT	3-(4,5-dimethyl-2-thiazolyl)-2,5-diphenyl-2-H-tetrazolium bromide
HepG2	Human hepatocellular carcinomas
TIC	Total ion chromatogram
MEP	Methyl esters product
SW480	Colon cancer
HGC	Human gastric cancer
DMSO	Dimethyl sulfoxide
DMEM	Dulbecco’s modified eagle medium
