# Supplementary material for: Extraction, Purification, and Elucidation of Six Ginkgol Homologs from Ginkgo biloba Sarcotesta and Evaluation of Their Anticancer Activities
Source: Molecules. 2022 Nov 11;27(22):7777. doi: 10.3390/molecules27227777 (PMC9699512; doi:10.3390/molecules27227777)
Supplement: Supplementary file 1 [file molecules-27-07777-s001.zip › molecules-1970013-supplementary.pdf]

Supplementary materials

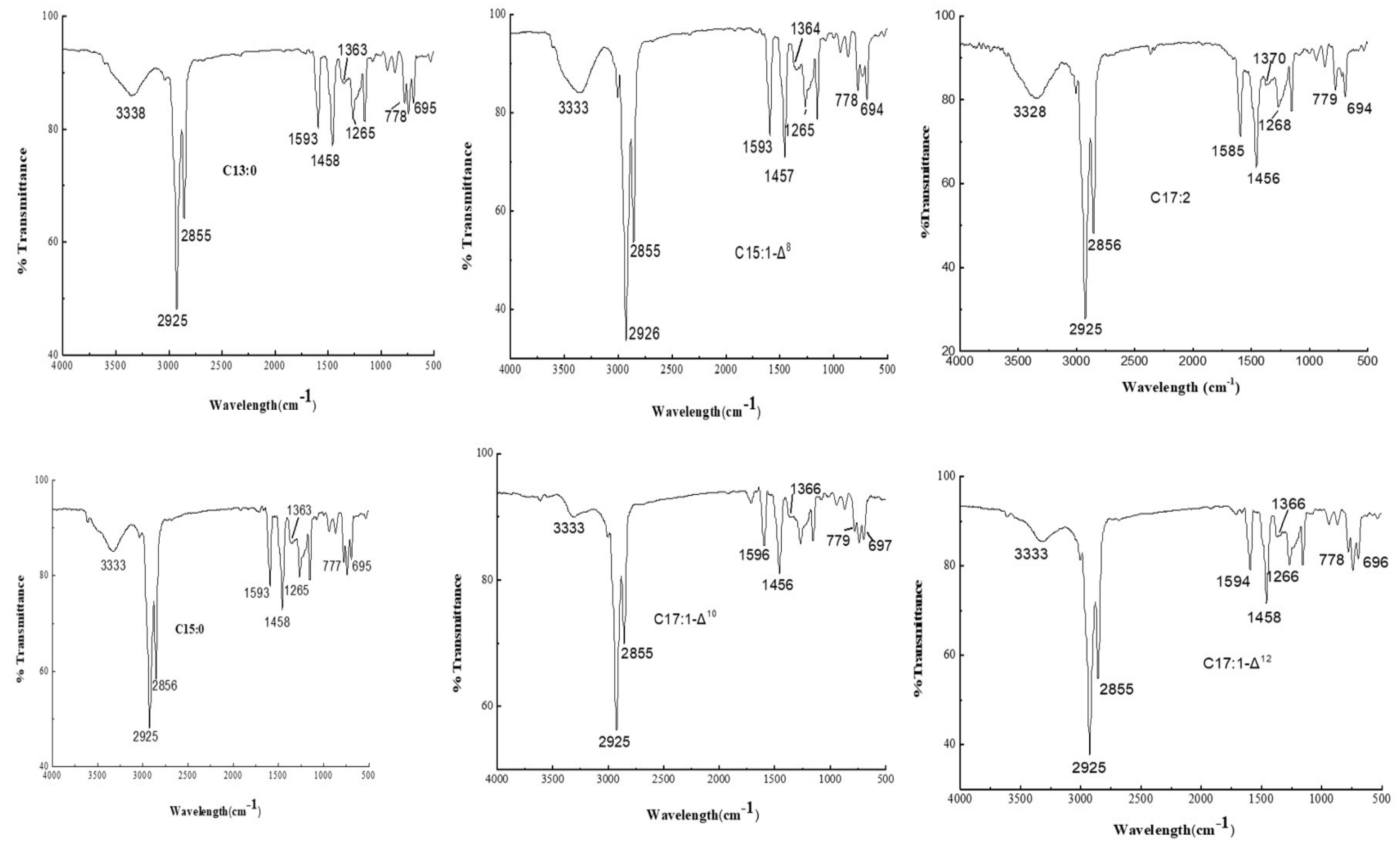

Figure S1. FTIR spectra of six ginkgols.

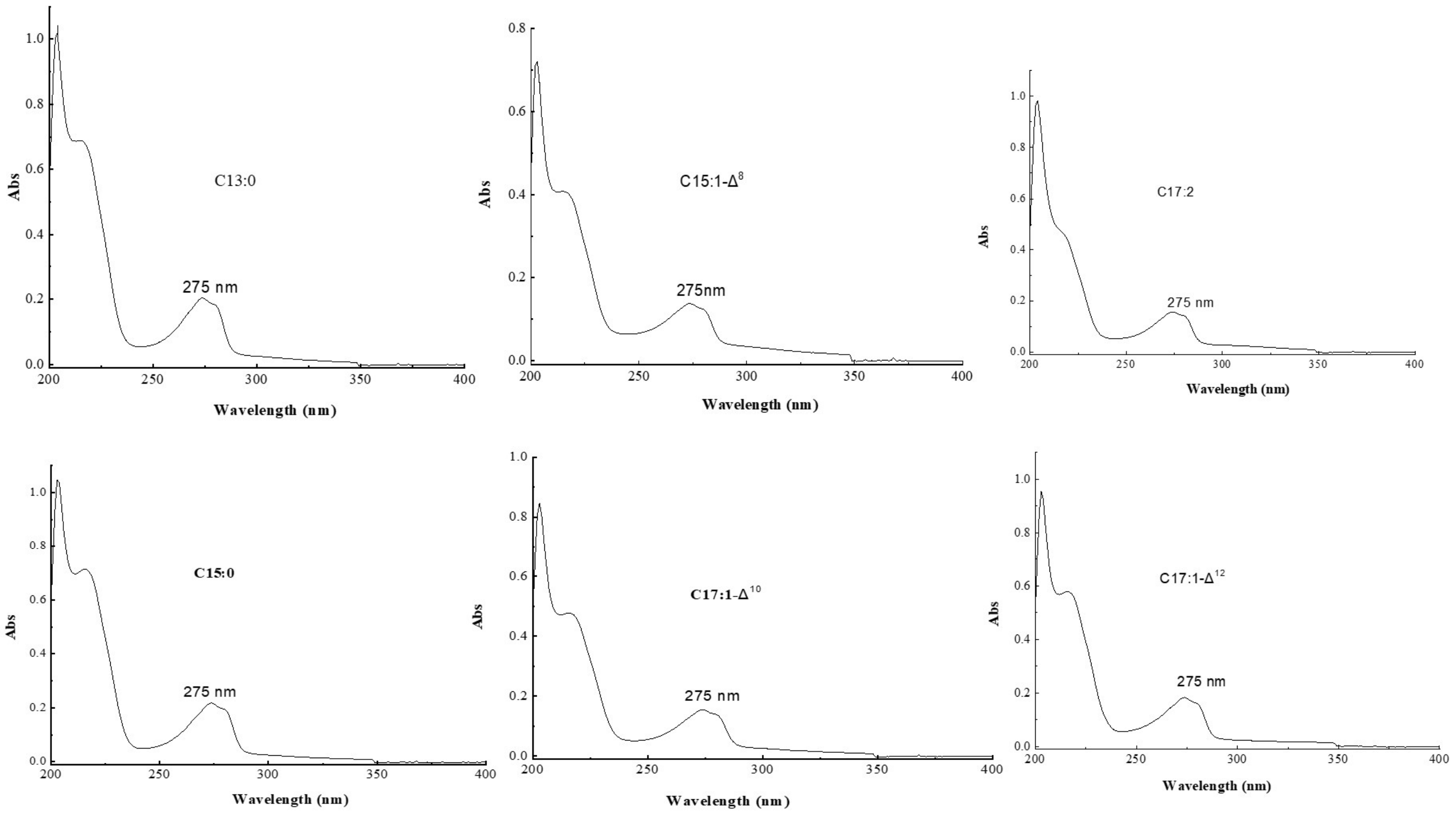

Figure S2. UV spectra of six ginkgols.

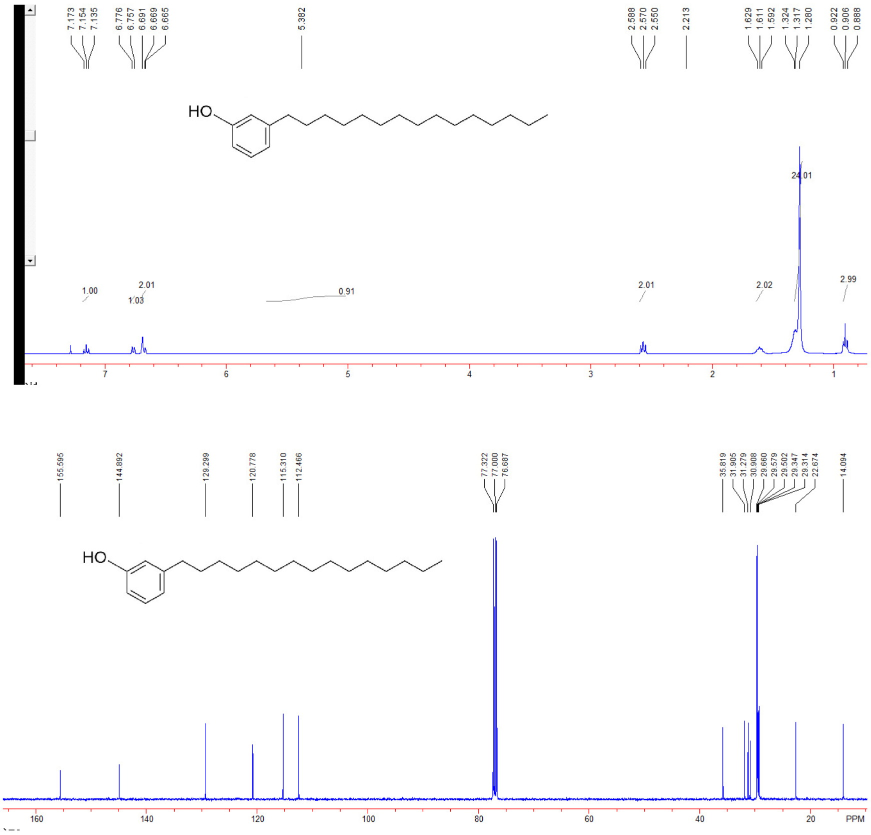

Ginkgol C15:0

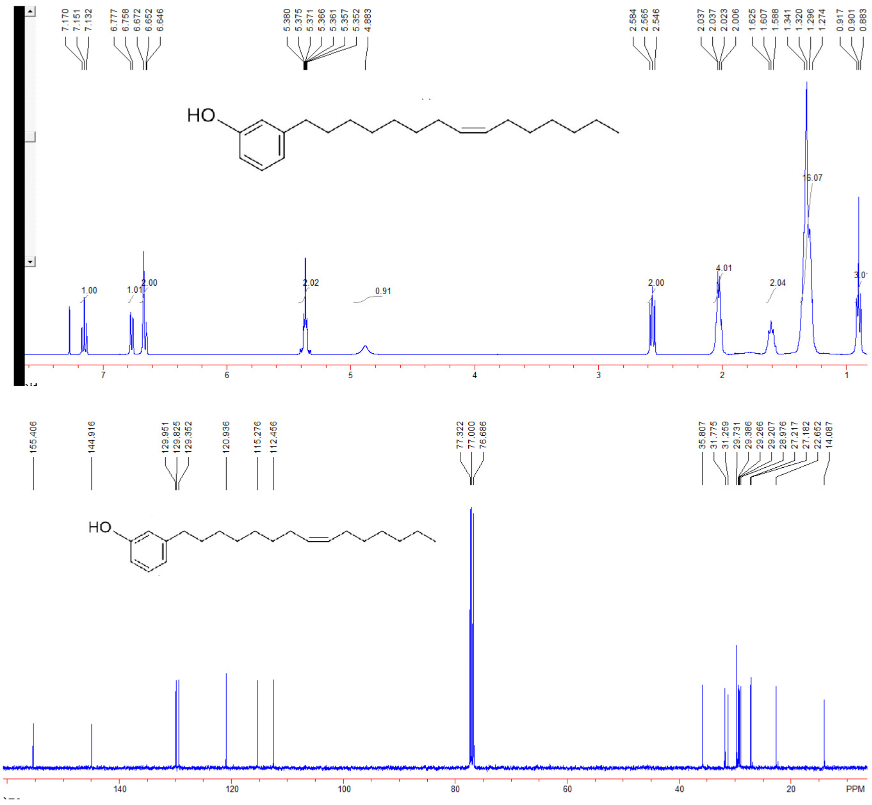

Ginkgol C15:1

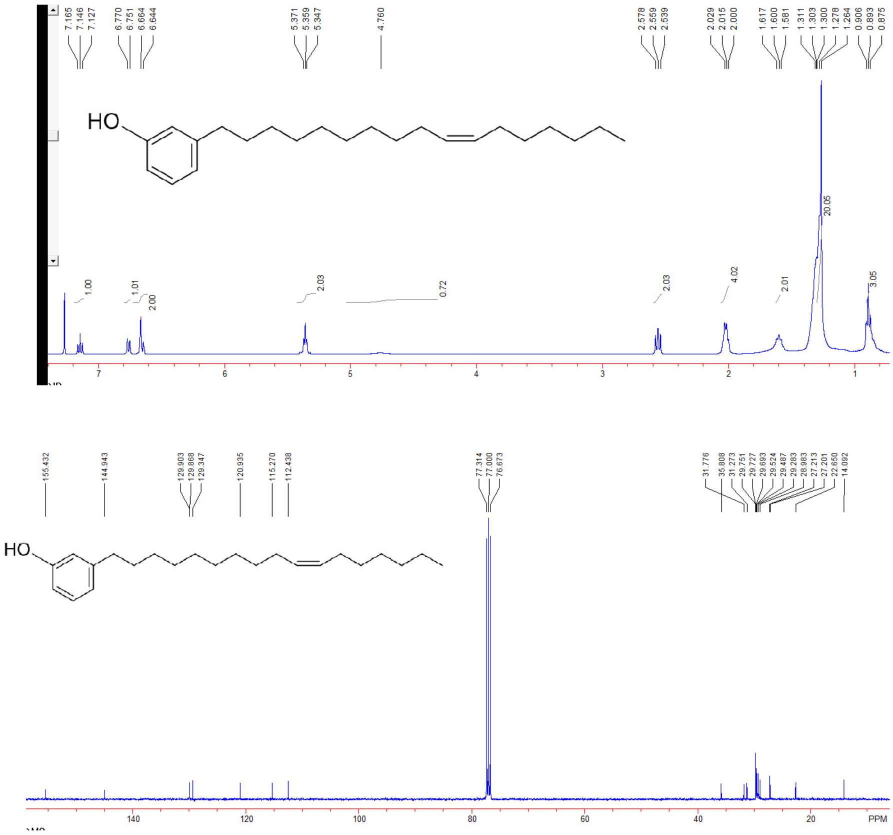

Ginkgol C17:1-Δ^{10}

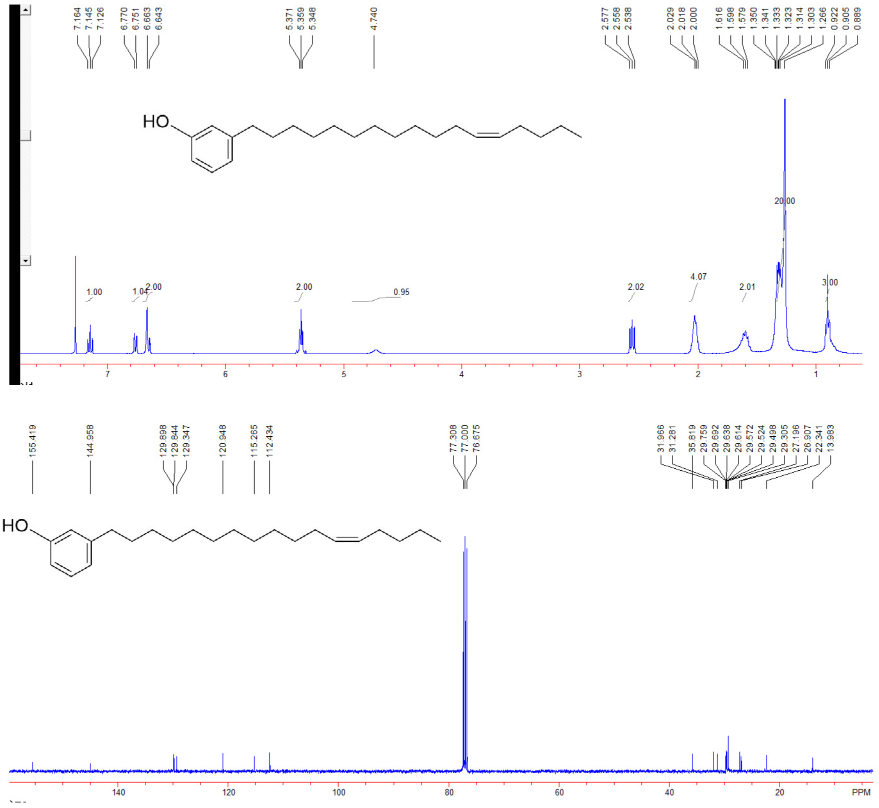

Ginkgol C17:1-Δ^{12}

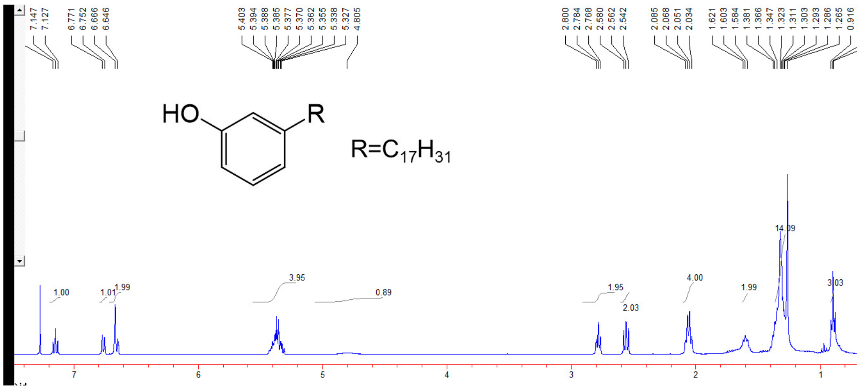

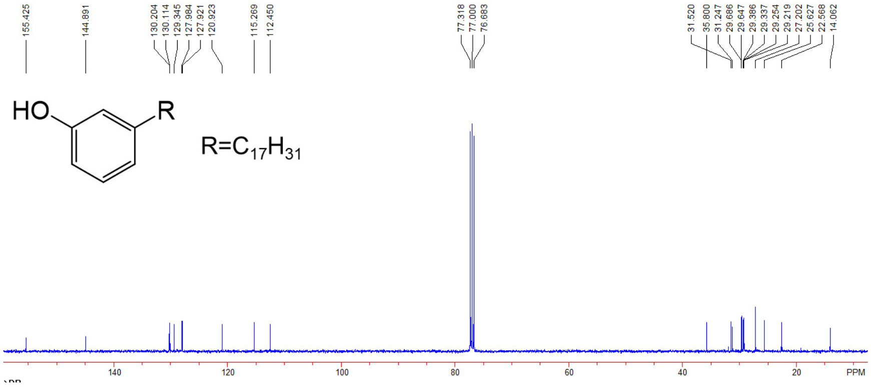

Ginkgol C17:2

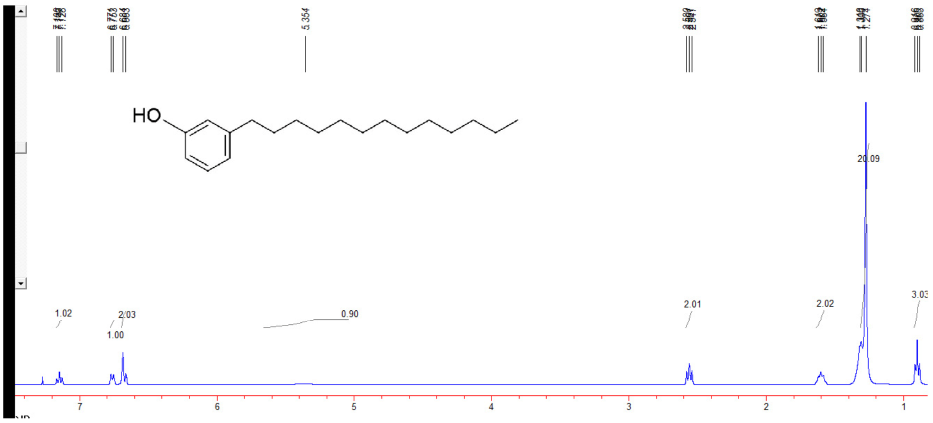

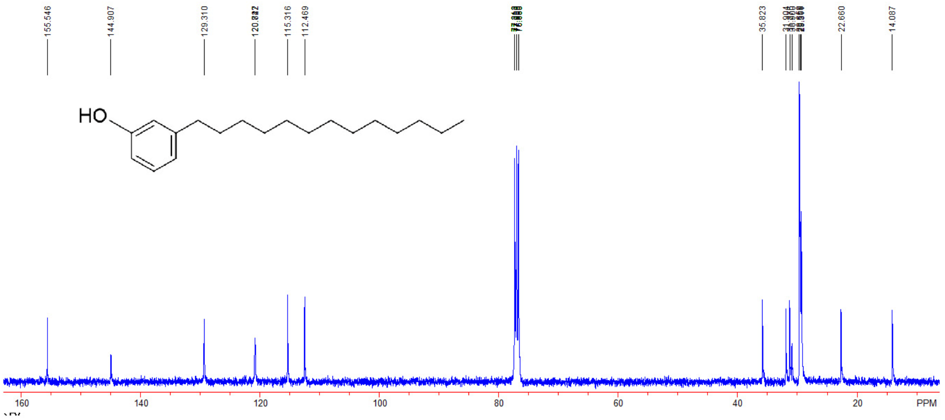

Ginkgol C13:0

Figure S3. ^{1}HNMR, and ^{13}CNMR spectra of six ginkgols.

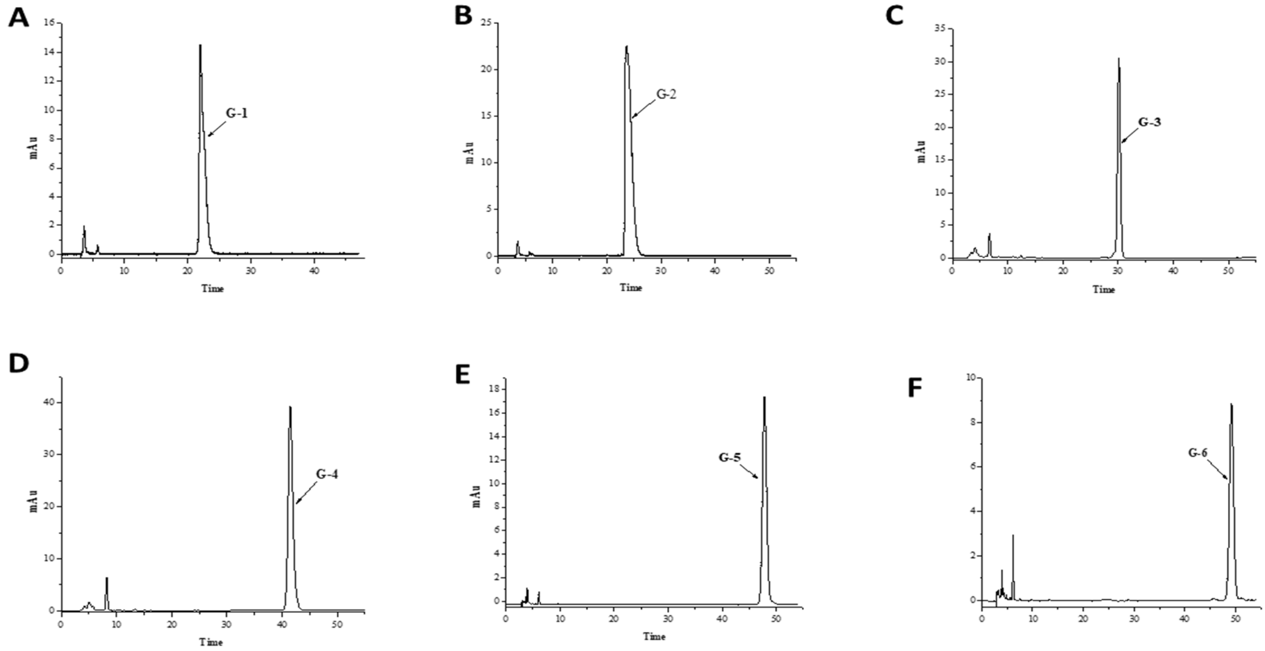

Figure S4. The chromatograms of the various ginkgols G-1, G-2, G-3, G-4, G-5, and G-6.

Table S1. Molarity of six ginkgols for anticancer activity assay

	Ginkgol＆Molecular weight					
	C13:0 (276)	C15:1-Δ^8 (302)	C17:2 (328)	C15:0 (304)	C17:1-Δ^{10} (330)	C17:1-Δ^{12} (330)
Concentration (μg/ml)	Corresponding molarity (μM)					
5	18.12	16.56	15.24	16.45	15.15	15.15
10	36.23	33.11	30.49	32.89	30.30	30.30
20	72.46	66.23	60.98	65.79	60.61	60.61
30	108.70	99.34	91.46	98.68	90.91	90.91
40	144.93	132.45	121.95	131.58	121.21	121.21
